# Identifying and Characterizing Alternative Molecular Markers for the Symbiotic and Free-Living Dinoflagellate Genus *Symbiodinium*

**DOI:** 10.1371/journal.pone.0029816

**Published:** 2012-01-04

**Authors:** Xavier Pochon, Hollie M. Putnam, Fabien Burki, Ruth D. Gates

**Affiliations:** 1 Hawai'i Institute of Marine Biology, School of Ocean and Earth Science and Technology, University of Hawai'i, Kane'ohe, Hawai'i, United States of America; 2 Department of Botany, University of British Columbia, Vancouver, British Columbia, Canada; Biodiversity Insitute of Ontario - University of Guelph, Canada

## Abstract

Dinoflagellates in the genus *Symbiodinium* are best known as endosymbionts of corals and other invertebrate as well as protist hosts, but also exist free-living in coastal environments. Despite their importance in marine ecosystems, less than 10 loci have been used to explore phylogenetic relationships in this group, and only the multi-copy nuclear ribosomal Internal Transcribed Spacer (*ITS*) regions 1 and 2 have been used to characterize fine-scale genetic diversity within the nine clades (A–I) that comprise the genus. Here, we describe a three-step molecular approach focused on *1*) identifying new candidate genes for phylogenetic analysis of *Symbiodinium* spp., *2*) characterizing the phylogenetic relationship of these candidate genes from DNA samples spanning eight *Symbiodinium* clades (A–H), and *3*) conducting in-depth phylogenetic analyses of candidate genes displaying genetic divergences equal or higher than those within the *ITS-2* of *Symbiodinium* clade C. To this end, we used bioinformatics tools and reciprocal comparisons to identify homologous genes from 55,551 cDNA sequences representing two *Symbiodinium* and six additional dinoflagellate EST libraries. Of the 84 candidate genes identified, 7 *Symbiodinium* genes (*elf2*, *coI*, *coIII*, *cob*, *calmodulin*, *rad24*, and *actin*) were characterized by sequencing 23 DNA samples spanning eight *Symbiodinium* clades (A–H). Four genes displaying higher rates of genetic divergences than *ITS-2* within clade C were selected for in-depth phylogenetic analyses, which revealed that *calmodulin* has limited taxonomic utility but that *coI*, *rad24*, and *actin* behave predictably with respect to *Symbiodinium* lineage C and are potential candidates as new markers for this group. The approach for targeting candidate genes described here can serve as a model for future studies aimed at identifying and testing new phylogenetically informative genes for taxa where transcriptomic and genomics data are available.

## Introduction

Dinoflagellates are a diverse group of single-celled eukaryotic algae that occur in marine and freshwater environments around the globe. They are ecologically important and serve as prominent primary producers in both their free-living [1], and symbiotic states [2]. Among their many unique attributes [3], dinoflagellates present a number of exceptional genetic characteristics, including nuclear chromosomes that remain condensed throughout the cell cycle [4], lack of conventional organization of DNA into histone-containing nucleosomes [5], and the presence of 5-hydroxymethyluracil in place of thymine [6]. In peridinin-containing species, there is also a plastid genome composed of a number of individual circular molecules of 2–3 kb which usually bear a single gene [7]. Studies of dinoflagellate gene structure have revealed the presence of many genes with high copy number and arrangement in polycistronic or otherwise tandem arrays [8], [9], a common feature that accounts for their enormous genome sizes (∼2–200 pg DNA per cell; [10], [11]). While such genome sizes have to date impeded attempts at comprehensive sequencing, an increasing number of nucleic acid sequences are available in the form of RNA transcripts, or expressed sequence tags (ESTs) and these datasets constitute a valuable comparative framework for examining dinoflagellate gene regulation, function, diversity and evolution [12]–[14].

The majority of symbiotic dinoflagellates are members of the diverse genus *Symbiodinium*. These photosynthetically active symbionts are crucial components of coral reef ecosystems and have been documented in a broad range of marine organisms, including ciliates, foraminifera, radiolarians, flatworms, anemones, zoanthids, jellyfish, corals, and mollusks [15]. In healthy scleractinian corals, *Symbiodinium* cells are typically found in high densities within the host tissues (>10^6^ cells cm^−2^; [16]) and provide the coral with essential sugars and amino-acids that allow them to sustain high rates of calcification. The host, in turn, provides *Symbiodinium* with a high light environment, as well as inorganic nitrogen and carbon [2]. Nine lineages of *Symbiodinium* spp., referred to as clades A through I [17], have been delineated phylogenetically using nuclear 28S (*nr28S*) and chloroplast 23S (*cp23S*) ribosomal DNA (rDNA). Each clade is further divided into multiple genetic strains that exhibit distinctive host taxonomic, geographic, and/or environmental distribution patterns, based on the highly variable nuclear internal transcribed spacer (*ITS-1* and *ITS-2*) regions of the rDNA operon [18]–[20]. The ITS region is currently most often utilized to resolve *Symbiodinium* diversity within each clade [21]–[23] and *ITS-2* has recently been used to designate species [24], [25], despite the fact that there is considerable variation among the copies of this gene found in individual genomes, a trait that complicates interpretation and makes species assignment problematic [26], [27].

To date, only a limited number of protein-coding genes have been used in a phylogenetic context for *Symbiodinium*. These include the coding region of the plastid-encoded photosystem II protein D1 (*psbA*; [28]), mitochondrial-encoded cytochrome oxidase 1 (*coI*; [29]), and cytochrome oxidase B (*cob*; [30], [31]), markers that have been applied to *Symbiodinium* strains belonging to five, six and two distinct clades, respectively. Overall, these studies reveal similar cladal evolutionary patterns between nuclear, chloroplastic, and mitochondrial genes [28], [29]. Other functionally important *Symbiodinium* genes, such as ribulose-1,5-bisphosphate carboxylase/oxygenase (*rbcL*; [8]), a 33 kDa peridinin-chlorophyll a-binding protein (*pcp*; [32]), and glyceraldehyde-3-phosphate dehydrogenase (*gapdh*; [33]), are complex multi-copy gene groups that show evidence of loci duplication, diverse isoforms, and potential lateral gene transfer, respectively. More recently, *actin* sequence information and copy-number estimates using quantitative PCR (qPCR) have become available for *Symbiodinium* [34], [35].

In general, molecular markers that distinguish groupings at the taxonomic scale of clade are conserved genes that are either too conserved to resolve, or have never been used to distinguish diversity within clades. In contrast, markers such as *ITS-2* and *actin*, which are variable enough to explore intraclade diversity, are multicopy genes that can be intragenomically variable, a facet that complicates interpretation. There is a clear need to develop new molecular markers that have the capacity to distinguish intraclade diversity in *Symbiodinium* and that meet the expectation of one sequence per *Symbiodinium* cell. This is particularly important when one considers that many Pacific corals harbor mixed *Symbiodinium* communities from one clade - clade C (e.g., [36]). In such cases, distinguishing between intragenomic variants and biological entities is important to understanding the taxonomic complexity of these interactions. Clade C also contains the greatest *ITS-2* sequence diversity and poses the most significant challenge in designating statistically supported, ecologically distinct species [26].

**Figure 1:**
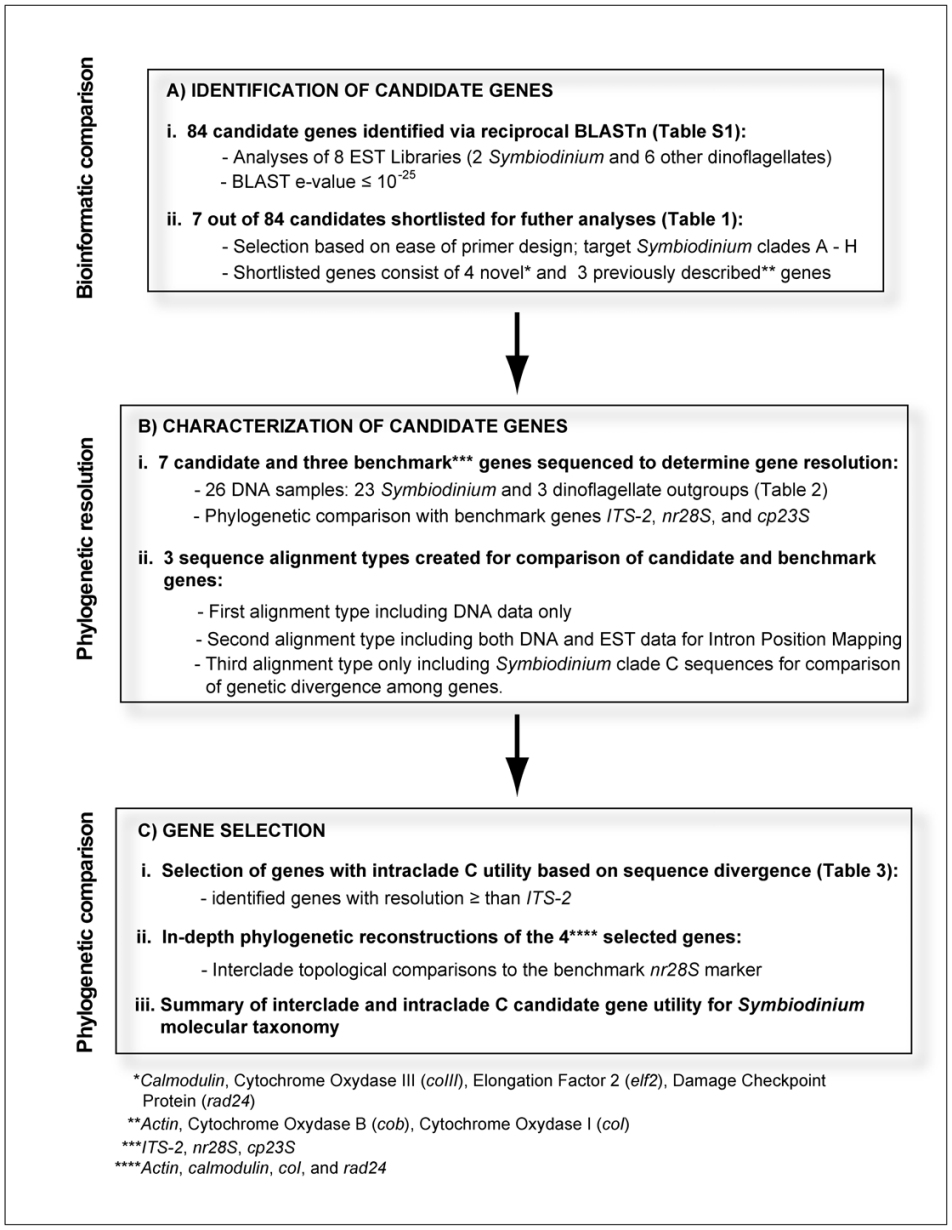
Flow diagram of the step-wise procedure for molecular marker identification.

In the spirit of improving capacity to examine *Symbiodinium* diversity in the context of coral biology, the goal of this study was to identify novel, and potentially single copy molecular markers that exhibit sufficient sequence divergence to distinguish *Symbiodinium* subclade types, particularly in clade C. To this end, we exploited bioinformatics tools and EST sequence data from *Symbiodinium* clade A [37], *Symbiodinium* clade C [38] and six non-*Symbiodinium* dinoflagellates [39]–[43]. Including *Symbiodinium* clades A and C as well as other dinoflagellates, brackets the genetic divergence in the genus to facilitate marker identification across all clades. Specifically our goals were to (*A*) identify novel *Symbiodinium* candidate genes using BLASTn comparisons of eight dinoflagellate EST libraries, (*B*) characterize the relationship between the sequences of these candidate genes amplified from DNA samples spanning the taxonomic diversity within *Symbiodinium*, and (*C*) conduct in-depth phylogenetic analyses of candidate genes displaying genetic divergences equal or higher than those within *ITS-2* of *Symbiodinium* clade C (Figure 1).

## Results

### 1. Identification of candidate genes

We targeted eight dinoflagellates EST libraries and used a bioinformatics approach [44] to identify *Symbiodinium* genes potentially useful as phylogenetic markers (Figure 1A, see methods for description of EST libraries). Reciprocal EST BLASTn comparisons were conducted between the two *Symbiodinium* EST libraries (clade A and C), and the six other dinoflagellate EST libraries (*Amphidinium carterae*, *Alexandrium tamarense*, *Heterocapsa triquetra*, *Karenia brevis*, *Karlodinium micrum*, and *Lingulodinium polyedrum*) in a pairwise fashion. This analysis identified 84 putative *Symbiodinium* genes with hits in two or more EST libraries at an e-value threshold of 10^−25^ (Table S1). The expression level of *Symbiodinium* transcripts ranged between 1 and 136. *Symbiodinium* EST A and C libraries shared ∼37% of orthologous genes (31 out of 84 genes) and almost all (30) of these were also shared with one or more of the non-*Symbiodinium* dinoflagellate EST libraries. The majority of the reciprocal BLASTn hits shown in Table S1 came from the *Symbiodinium* clade C library (63% of the 84 putative *Symbiodinium* genes) reflecting the greater number of ESTs in this library (5,156 versus 2,163 ESTs in clade A).

Of the 84 candidate genes identified via BLAST analyses (BLASTn and BLASTx), seven were selected for downstream analyses (Table 1; Table S1). The remaining 77 were deemed unsuitable for further analysis because the alignments revealed high levels of variation among sequences that precluded primer design. Of the seven taken forward, elongation factor 2 (*elf2*), cytochrome oxydase III (*coIII*), *calmodulin* (abbreviation for CALcium-MODULated proteIN), and damage checkpoint rad24 protein (*rad24*) have not been applied to *Symbiodinium* before, while cytochrome oxidase I (*coI*), *actin*, and cytochrome B (*cob*) have been partially characterized for *Symbiodinium* [29], [31], [35].

**Table 1:** Seven out of eighty-four candidate genes selected for downstream analyses.

Protein description	# EST	Libraries Hits [e-value≤10^−25^]
Cytochrome oxidase subunit 3 (*coIII*)	35	A, C, At, Kb, Km, Lp
*Actin*	19	A, C, Ac, At, Ht, Kb, Km, Lp
DNA damage checkpoint protein (*rad24*)	10	A, C, At, Km
Mitochondrial cytochrome B (*cob*)	8	A, C, Kb, Km, Lp
*Calmodulin*	8	C, Ac, At, Ht, Km, Kb, Lp
Cytochrome oxidase subunit 1 (*coI*)	4	A, C, Ac, Kb, Km, Lp
Translation elongation Factor 2 (*elf2*)	4	A, C, At, Km

Genes are sorted by decreasing level of transcript abundance in the two *Symbiodinium* EST libraries combined. Details of the EST libraries displaying hits in BLASTn are indicated for each gene by letters A, C, Ac, At, Ht, Kb, Km, and Lp, which correspond to EST libraries *Symbiodinium* A, *Symbiodinium* C, *Amphidinium carterae*, *Alexandrium tamarense*, *Heterocapsa triquetra*, *Karenia brevis*, *Karlodinium micrum*, and *Lingulodinium polyedrum*, respectively. Protein descriptions were obtained using BLASTx. The complete list of candidate genes (n = 84) identified after BLASTn comparisons of eight dinoflagellate EST libraries is presented in Table S1.

### 2. Characterization of candidate genes

The goal of this step was to examine the seven gene candidates selected for downstream analysis for presence/absence of introns and rates of average genetic divergence within *Symbiodinium* lineage C (Figure 1B), as well as to generate sequences for phylogenetic analyses. To provide a comparative framework, we also examined three benchmark genes; the nuclear Internal Transcribed Spacer 2 region (*ITS-2*) and large subunit ribosomal D1–D3 region *(nr28S)*, and the chloroplast large subunit ribosomal DNA domain V (*cp23S*), in addition to the seven gene candidates. All ten genes were amplified from DNAs representing 23 *Symbiodinium* samples spanning eight clades (A–H) and three closely related dinoflagellates (*Gymnodinium simplex*, *Pelagodinium beii*, and *Polarella glacialis*) (See Table 2 for details of DNAs). This effort generated high-quality direct sequences for four genes (*elf2*, *coI*, *coIII*, and *cob*) and unreadable sequences, or numerous base-pair ambiguities for the remaining three gene candidates (*calmodulin*, *rad24*, and *actin*) and the three benchmark genes. The amplified products of these six genes were therefore cloned and ≤8 bi-directional sequences per DNA and gene produced. In full, between 21 and 92 DNA sequences per gene were generated, representing a total of 485 assembled gene sequences. The candidate genes were amplified successfully from the majority of the 23 *Symbiodinium* DNAs and only 10 PCR reactions failed to amplify (4.8%; data not shown). Individual sequence alignments, including the *Symbiodinium* and outgroup samples, were created for each gene under investigation (DNA sequence alignments available upon request). Eight sequences (six *rad24* and two *actin* sequences, i.e., 1.65%) of the 485 sequences retrieved were identified as potential chimeras by the software package Bellerophon [45], and were removed from downstream analyses. The subclade identity of each *Symbiodinium* sample (Table 2) was determined by locally blasting our *ITS-2* sequence alignment benchmark against the global *ITS-2* database available in GeoSymbio version 1.0.1 [46]. All but four *Symbiodinium* samples matched previously published *ITS-2* sequence types with 100% BLASTn homology. Four new sequence types, differing from sequence types B19, F5.1, F5.2e, and G2, by 2 to 5 bp received the new *ITS-2* names B19a, F5.1d, F5.2g, and G2b, respectively. All sequences were submitted to GenBank and assigned the following accession numbers: JN558040–JN558110 (*ITS-2* and *nr28S*), JN557969–JN558039 (*cp23S*), JN557869–JN557890 (*elf2*), JN557891–JN557916 (*coI*), JN557917–JN557942 (*coIII*), JN557943–JN557968 (*cob*), JN558111–JN558202 (*calmodulin*), JN558203–JN558275 (*rad24*), and JN558276–JN558346 (*actin*). Phylogenetic reconstructions of two of the benchmark genes (*nr28S* and *cp23S*) using the model of evolution and parameters shown in Table S2, visualized the evolutionary relationships between the DNA samples investigated (Figure S1) and resulted in identical topologies to those published previously [17], [47].

**Table 2:** Description of the genomic DNAs used in this study.

Name^a^	ITS2^b^	Host origin	Culture^c^	GenBank	Reference
A	A2_1	*Bartholomea annulata*	RT-23	JN558097	This study
A	A2_2	*Gorgonia ventallina*	RT-89	JN558100	This study
A	A3	*Pseudoplexaura porosa*	725	JN558091	This study
A	A13	*Plexaura kuna*	708	JN558094	This study
B	B1	*Plexaura kuna*	704	JN558057	This study
B	B2	*Eunicea flexuosa*	Pflex	JN558060	This study
B	B19a*	*Plexaura kuna*	703	JN558055	This study
C	C1	*Amphisorus hemprichii*	N/A	AM748551	[71]
C	C15	*Amphisorus hemprichii*	N/A	AM748552	[71]
C	C90	*Sorites* sp.	N/A	AM748554	[71]
C	C91	*Sorites* sp.	N/A	AM748555	[71]
D	D1	*Acropora* sp.	A001	JN558075	This study
D	D1a	unknown anenome	Ap02	JN558078	This study
D	D1.2	*Haliclona koremella*	PSP1-05	AM748617	[71]
E	E1	*Anthopleura elegantissima*	RT-383	JN558084	This study
F	F1	*Montipora verrucosa*	Mv	JN558066	This study
F	F5.1	*Meandrina meandrites*	RT-133	AM748592	[71]
F	F5.1d*	*Sinularia* sp.	Sin	JN558069	This study
F	F5.2g*	*Montastraea faveolata*	Mf	JN558072	This study
G	G2	*Marginopora vertebralis*	N/A	AM748598	[71]
G	G2b*	*Marginopora vertebralis*	N/A	JN558087	This study
H	H1	*Sorites* sp.	N/A	AM748602	[71]
H	H1a	*Sorites* sp.	N/A	AM748603	[71]
*G. simplex*	N/A	N/A	CCMP419	JN558103	This study
*P. beii*	N/A	N/A	PB-1	JN558106	This study
*P. glacialis*	N/A	N/A	CCMP1383	JN558108	This study

a Letters A to H correspond to the *Symbiodinium* clades. Species names of outgroup samples are indicated: *Gymnodinium simplex*, *Pelagodinium beii*, and *Polarella glacialis*.

b Alpha-numeric names correspond to *Symbiodinium ITS-2* rDNA molecular taxonomy sensu [71]. Letters correspond to the *Symbiodinium* clades, and numbers correspond to a specific *ITS-2* sequence. All samples are genetically distinct, except for *Symbiodinium* A2, which was found in two distinct cultures and referred here to as A2_1 and A2_2. Sample D1.2 corresponds to the PSP1-05 sample originally isolated from the sponge *Haliclona koremella* (see [71] for details).

c Culture names of DNAs extracted from *Symbiodinium* cultures. N/A = Not Available.

*Indicates new sequences.

**Figure 2:**
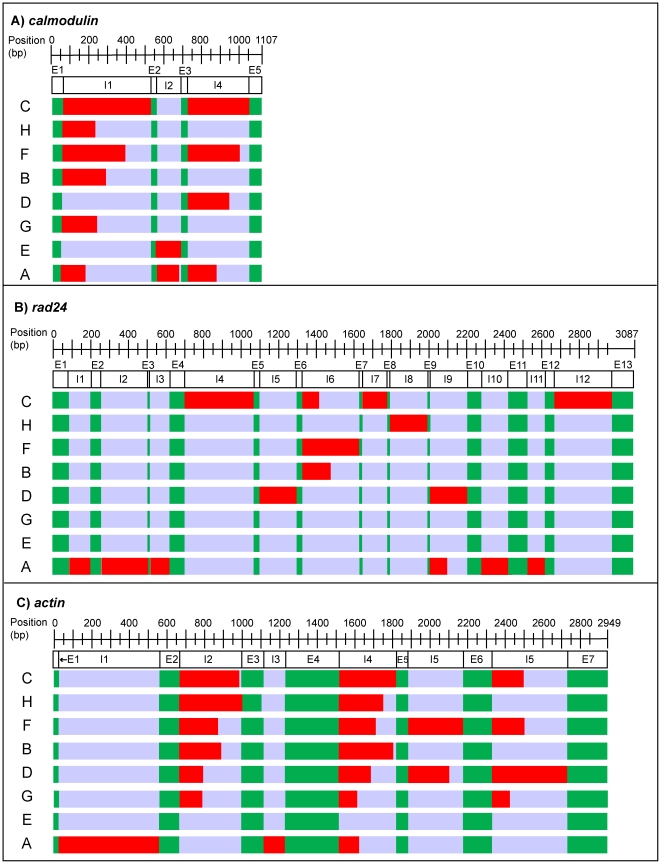
Intron position mapping of three intron-containing genes. Positions and numbers of coding (exons [E]; shown in green) and non-coding regions (introns [I]; shown in red) in the genes *calmodulin* (**A**), *rad24* (**B**), and *actin* (**C**). The sizes of the non-coding regions indicated here depict the maximum intron size recorded in genomic samples in each *Symbiodinium* clade. DNA alignments ranged from 1,107 bp to 3,087 bp in length and letters A to H correspond to the eight *Symbiodinium* clades.

A second set of sequence alignments comprising sequences amplified from genomic DNAs and originating from the EST libraries were used to identify the number and position of introns. *Calmodulin*, *rad24*, and *actin* contained highly variable introns that increased the lengths of alignments from ∼150 to 1,107 bp, ∼600 to 3,087 bp, and ∼900 to 2,949 bp, as compared to corresponding EST alignments, respectively (Figure 2). Between 3 (*calmodulin*) and 12 (*rad24*) introns were identified and some intron positions appeared unique to a given clade, whereas others were shared among clades. For example, the *rad24* intron positions I1–I3 are unique to *Symbiodinium* clade A, while I4 and I12 are restricted to clade C (Figure 2B). In contrast, intron positions I2 and I4 of *actin* are shared between the six terminal *Symbiodinium* clades G, D, B, F, H, and C (Figure 2C). In some cases, introns appeared so variable that the alignment of sequences among and even within some clades was impossible, reflecting the fact that they were not the same length and/or shared by all *Symbiodinium* types within a clade.

The third set of alignments comprised only clade C sequences and were constructed to allow for a comparison of within clade C divergence rates for each gene and to identify candidates with evolutionary rates equal to or higher than the *ITS-2*. The *ITS-2* locus displayed an average genetic distance of approximately 1%, which constituted the benchmark here. The average uncorrected genetic distance between clade C sequence types ranged between 0.24% in *cp23S* to 22.49% in *calmodulin* (Table 3). The genetic divergences for *elf2*, *coIII*, and *cob* were below 1%, and for *coI* were approximately equivalent to *ITS-2* at 1.13%. *Calmodulin*, *rad24*, and *actin* all displayed significantly different genetic divergence rates depending on whether introns were excluded or included from the calculations. *Actin* was similar to *ITS-2* when the introns were excluded (1.07%) but 4.16% when the introns were included. Values for *rad24* were higher, with 1.94% and 11.69% of genetic distance without and with introns, respectively. The highest genetic divergences were recorded in *calmodulin*, with values of 4.7% and 22.49% excluding and including the introns, respectively.

**Table 3:** Estimation of divergence rates between markers for *Symbiodinium* types C1, C15, C90, and C91.

	*ITS-2*	*nr28S*	*cp23S*	*elf2*	*coI*	*coIII*	*cob*
**Min**	0.35	0.25	0	0.21	0.15	0	0.33
**Max**	1.8	0.63	0.49	0.86	2.12	1.29	1.12
**Average**	0.95	0.38	0.24	0.57	1.13	0.72	0.76
**Variance**	1.05125	0.0722	0.12005	0.21125	1.94045	0.83205	0.31205

	*calmodulin* (−)	*calmodulin* (+)	*rad24* (−)^a^	*rad24* (+)^a^	*actin* (−)^b^	*actin* (+)^b^
**Min**	3.4	5.3	N/A	N/A	0.32	1.39
**Max**	6.5	31.84	1.94	11.69	1.5	5.78
**Average**	4.7	22.49	1.94	11.69	1.07	4.16
**Variance**	4.805	352.1858	N/A	N/A	0.6962	9.63605

The minimum, maximum and averaged uncorrected genetic distances among clade C *Symbiodinium* types are indicated for each marker investigated. Calculations for *calmodulin*, *rad24*, and *actin* were made on sequence alignments excluding (−) and including (+) introns.

a No sequences were obtained for *Symbiodinium* C90 and C91 so only two types were compared.

b No sequences were obtained for *Symbiodinium* C90, so only three types were compared.

### 3. Gene Selection

The four candidate genes (*coI*, *calmodulin*, *rad24*, and *actin*) with average genetic divergences higher than *ITS-2* were selected for in-depth phylogenetic analyses, with the goal of comparing the evolutionary relationships among *Symbiodinium* clades as well as among genes, and to test the utility of each gene for future investigations of *Symbiodinium* molecular taxonomy, particularly within *Symbiodinium* clade C (Figure 1C). Phylogenetic reconstruction of *coI* was based on the genomic sequence alignment (1057 bp), and on exon regions only for *calmodulin* (154 bp), *rad24* (580 bp), and *actin* (925 bp).

**Figure 3:**
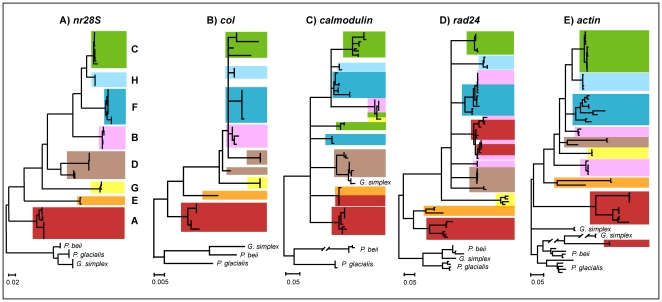
Topological comparison of benchmark *nr28S* and four selected candidate genes. (**A**) 71 nuclear large subunit (*nr28S*) sequences (alignment size: 915 bp) (**B**) 26 cytochrome oxidase subunit 1 (*coI*) sequences (1057 bp), (**C**) 92 *calmodulin* sequences (154 bp), (**D**) 73 *rad24* sequences (580 bp), and (**E**) 71 *actin* sequences (925 bp). The *nr28S* topology is used here as the benchmark marker, with colors corresponding to clades A (red), B (pink), C (green), D (brown), E (orange), F (dark blue), G (yellow), and H (light blue). Phylogenies are rooted using the dinoflagellates *Gymnodinium simplex*, *Pelagodinium beii*, and *Polarella glacialis*. Detailed phylogenetic reconstructions, including node support values from the ML bootstrap analyses and Bayesian posterior probabilities, as well as the GenBank accession numbers, are shown in Figures S1, S2, S3, S4, S5.

The topology of the resulting trees was compared to the *nr28S* gene phylogeny as the benchmark for clade level relationships (Figure 3). Overall, *Symbiodinium* clade arrangements were relatively similar between genes, particularly with respect to the position of clades A and E and the derived positions of clades B, F, H, and C. The phylogenetic reconstruction of the *coI* gene, which extends the previous work from Takabayashi et al. [29] by including representatives in clades G and H, was most similar to our benchmark *nr28S* gene despite an obvious lack of support for the node between terminal clades D, B, F, H, and C (Figure 3B; Figure S2). Interclade relationships were also unsupported for the majority of intron-containing genes. For example, *calmodulin* and *rad24* produced completely unresolved topologies, which was expected given the short lengths and variability of the gene fragments analyzed (Figure 3C and 3D). The *actin* gene, however, yielded a relatively well-resolved topology with *Symbiodinium* clades that corresponded roughly to the *nr28S* topology (Figure 3E).

A number of paralogous copies and/or samples deviating significantly from the expected phylogenetic positions (*sensu nr28S* topology) were observed for all three intron-containing genes. For example, the *calmodulin* topology revealed the presence of paralogous copies in *Symbiodinium* clades C and F, specifically sample C1 and F5.2g. Additionally, other *calmodulin* samples deviated significantly from their expected phylogenetic positions, including samples in clades A, C, and G (samples A2_1, C91, and G2) and the outgroup sample *Gymnodinium simplex* which grouped within clade D (Figure 3C; Figure S3). For *rad24*, paralogous copies were found in *Symbiodinium* clades A and B (samples A2_1, A3, B2, and B19a), with sample B1 resolving in clade F (Figure 3D; Figure S4). Finally, the *actin* gene also displayed a number of paralogous copies in *Symbiodinium* clades A, B, and F (samples A2_2, B1, F5.1, and F5.2g), as well as a sample in clade B (sample B19a) that deviated from its expected phylogenetic position (Figure 3 E; Figure S5).

## Discussion

Our knowledge of *Symbiodinium* diversity has been constrained by the limited number of phylogenetic markers that have been applied to this group. To date, nine loci (nuclear *18S*, *ITS-1*, *5.8S*, *ITS-2*, and *28S*; chloroplastic *23S*, and *psbA*; mitochondrial *cob* and *coI*) have been used to examine *Symbiodinium* diversity in a phylogenetic context [28], [29], [31], [36], and evolutionary relationships among *Symbiodinium* clades have never been inferred using more than two concatenated genes [47]. This contrasts with other research fields where more than 100 concatenated genes are commonly used to address key evolutionary questions [48]–[50]. Recently acquired EST data for dinoflagellates and the availability of *Symbiodinium* EST libraries [37]–[43] represent valuable sources of sequence information. These resources create the opportunity to identify new molecular markers that can expand our knowledge of the diversity, molecular taxonomy, and evolution in *Symbiodinium*.

### Identification of Candidate Genes: Utility of the bioinformatics approach

Prior studies have examined *Symbiodinium* EST libraries to determine how gene expression patterns in *Symbiodinium* clade C respond to stress [38], and to compare functional groups of genes among *Symbiodinium* clades A and C and other eukaryotes [37]. Here we used a bioinformatics approach [44] to retrieve homologous gene sequences from eight EST datasets comprising 55,551 dinoflagellate cDNA sequences. This analysis identified 84 candidate genes shared by at least two dinoflagellate libraries (Table S1). Of these 84 genes candidate only 7 (Table 1) allowed for the design of primer sets. This number was surprisingly low, reflecting a number of issues inherent in the EST libraries, including an imbalance in the number of ESTs in the clade A and clade C libraries as well as significant differences in environmental conditions under which the EST libraries examined were generated (see Materials and Methods).

The expression level of *Symbiodinium* genes, as derived by the observed number of *Symbiodinium* ESTs per gene, decreased rapidly among the 84 candidates, limiting the design of primers in most cases. For example, among the 31 orthologous genes shared between the two *Symbiodinium* libraries (Table S1), 7 were highly expressed (i.e., ≥10 ESTs), 8 moderately expressed (i.e., between 5 and 9 ESTs), and 16 displayed low expression (i.e., <5 ESTs). Primer design was particularly challenging in the last two categories, and impossible for those genes containing ≥3 *Symbiodinium* ESTs. Bachvaroff and Place [14] suggest that highly expressed dinoflagellate genes are more likely to be found in tandem repeats with low intron density and high copy number, whereas moderately to low expressed genes are by definition, less well represented in EST surveys and more likely to be single copy with a higher intron density. This hypothesis suggests that investigating candidate genes that are expressed at low levels may be most fruitful in the search for highly variable genes with low copy number. Here we were only able to target two candidate genes in the low expression category, a limitation that may have precluded us from selecting single copy genes for downstream analyses. In contrast, the Marine Microbioal Eukaryote Transcriptome Project is currently sequencing 750 eukaryotic microbe transcriptomes using next generation sequencing (NGS) technologies. The approved transcriptomes include 31 *Symbiodinium* samples from 10 strains that belong to 4 clades (A, C, D, and F; http://marinemicroeukaryotes.org/). These publicly available deep transcriptomes will greatly benefit and facilitate future studies aimed at applying a comparative bioinformatics approach to identifying *Symbiodinium* genes with phylogenetic relevance that cross a range of expression levels.

### Characterization of candidate genes: Insights from cDNA to genomic sequence data

DNA sequences were successfully generated for the seven candidate genes from the majority of *Symbiodinium* DNA samples, confirming that our primers were successful at targeting all clades within the genus. This effort makes a significant contribution to the field by generating DNA sequences for *Symbiodinium* samples from clades A–H for three partially characterized genes (*coI* [29]; *cob* [31]; *actin* [35]) and four novel genes (*elf2*, *coIII*, *calmodulin*, and *rad24*). Notably, this is the first study to generate DNA sequences for *Symbiodinium* clades A–H across the only three protein coding genes (*coI*, *coIII*, and *cob*) that constitute the highly reduced mitochondrial genome of dinoflagellates [51].

Comparing EST-and-genomic sequence alignments allows non-coding DNA regions or introns to be identified. These are hyper-variable regions that can provide fundamental evolutionary insights and are broadly used for finer-scale phylogenetic reconstructions of a wide range of taxa. A very limited number of studies have compared dinoflagellate cDNA with their genomic counterparts [14], [52], and much work remains to be done before we gain a full understanding of the evolution of introns among dinoflagellates genomes. Here, three of the nuclear genes (*calmodulin*, *rad24*, and *actin*) were found to contain from three (*calmodulin*) to twelve (*rad24*) variable intron regions (Figure 3). This number is high by comparison to non-symbiotic dinoflagellates where introns are not very common and intron numbers are relatively low in the few genes that have been analyzed [53]. For example, a recent survey shows that introns are present in only 3 of 17 heat shock protein genes sequenced; one canonical intron in *Peridinium willei* and *Thecadiniium yashimaense* and one non-canonical in *Polarella glacialis* [54]. In another study, 15 out of 47 genes (i.e., 32%) in *Amphidinium carterae* were found to contain introns [14], although the majority was found at low density (1–3 introns per gene). Remarkably, the latter study found only a single intron associated with the *actin* gene and concluded that one potential hallmark of dinoflagellates is that more highly expressed genes, such as *actin*, are intron-poor. Our analysis does not support this conclusion; the *Symbiodinium actin* gene contains six introns, comparable to the quantity found in mammalian genes (5–10 genes; [55]). In fact, among the four genes chosen for full phylogenetic analysis (*coI*, *calmodulin*, *rad24*, and *actin*), there was no correlation between the number of ESTs (the proxy for expression level); 4, 8, 10, and 19, respectively, and intron number; 0, 3, 12, and 6 respectively. Additional studies are required to determine whether the increased intron density observed in *Symbiodinium* is an evolutionary consequence of the symbiotic mode of life or reflects other evolutionary constraints.

*Actin* is often described as a complex multi-copy gene family. In multicellular animals, the phylogeny of the *actin* gene family relates to distinct functional categories such as smooth, cytosolic, or cardiac muscle actins [56]. Furthermore, many eukaryotes harbor an assemblage of more divergent *actin*-related proteins referred to as *arp1* to *arp11*, each with distinct functions [57]. In contrast, almost all canonical *actin* protein sequences in dinoflagellates vary slightly from copy to copy, and there seems to be no correlation of conservation and expression. For example, in the dinoflagellate *Dinophysis acuminata* and *Dinophysis caudata* [52] and *Amphidinium carterae* [14], the pool of total genomic *actin* copies includes variable gene copies and some apparent pseudogenes. As a result, Kim et al. [52] proposed the actin gene family in dinoflagellates best fit the “birth and death” model of evolution [58] based on recent duplications, pseudogenes, and incomplete lineage sorting.

Our in-depth genomic analysis of *actin* suggests that this gene is also complex in *Symbiodinium*. Our data show high variability in intron positioning, high sequence divergence among samples of a given clade (Figure 2), and evidence of paralogous copies within *Symbiodinium* clades A, B, and F. *Actin* has previously been used to quantify *Symbiodinium* cells in coral tissues using real-time PCR (rtPCR; [35]). The latter study described the use of *Symbiodinium* clade C-specific and clade D-specific rtPCR primers located within the intron I4 (see Figure 2C), and estimated the *actin* gene copy number per *Symbiodinium* cell to be relatively low and set at 7.0±2.9 (SD) and 0.98±0.66 (SD) for C and D, respectively. The comparison of our EST-to-genomic *actin* alignment with the rtPCR primer pairs used in Mieog et al. [35] reveals a number of issues with this interpretation. For example, the Mieog et al. [35] clade C primer sets only recognize our *Symbiodinium* C1 sequences but not C15 or C91 sequences, hence these primers are not clade C-specific, but rather subclade C1-specific. Also, the Mieog et al. [35] clade D primers do not match any of our clade D sequences, suggesting that they were either targeting a different *Symbiodinium* population (allelic variation), a specific isoform, or a distinct *actin* family gene within clade D. Our observations indicate that additional work is needed to characterize the gene structure and extent of intragenomic variation of the *Symbiodinium actin* gene, and to evaluate the usefulness of this highly-variable phylogenetic marker.

### Genes Selection: No one gene fits all

Our results collectively drive home the fact that no single gene fits all of the taxonomic questions that we have for the genus *Symbiodinium*. Prior studies have opted to use relatively conserved genes (*nr18S* or *nr28S*) to address specific questions related to host-symbiont associations, ecological distribution, and/or abundance of *Symbiodinium* clades among reef invertebrates and protists [59], [60]. Other investigators have targeted faster evolving genes (*cp23S*, *nrITS*, or even microsatellites) to address questions related to finer-scale (within-clade) patterns of specificity, biogeography and ecology [20], [21], [36], [61]. Because nuclear and chloroplastic ribosomal genes show very similar evolutionary histories, at both the clade level [17], and within-clade level [62] and because there is no published evidence of hybridization or differential introgression of nuclear and plastid genomes between clades, it is commonly assumed that each *Symbiodinium* clade is reproductively and evolutionarily isolated and that accurately deciphering *Symbiodinium* taxonomy at a finer-scale is simply a matter of targeting a faster evolving genes. Our analysis of average genetic divergences within clade C identified four faster evolving genes (*coI*, *calmodulin*, *rad24*, and *actin*) with potential utility for finer-scale taxonomic analysis within the genus. Our in-depth phylogenetic analyses of the coding regions of these genes, however, revealed considerable complexity in the behavior of each, suggesting that the rate of evolution is not the only criterion that is important in selecting new molecular markers. Below we discuss the unique characteristics and pros and cons of each of the four faster evolving genes we explored in detail here (summarized in Table 4).

**Table 4:** Characteristics of the four genes (*coI*, *calmodulin*, *rad24*, and *actin*) selected for in-depth phylogenetic analyses.

	*coI*	*calmodulin*	*rad24*	*actin*
**Presence of intron Regions**	No	Yes (3 introns)	Yes (12 introns)	Yes (6 introns)
**Presence of paralogous copies**	No	Yes (clades C and F)	Yes (clades A and B)	Yes (clades A, B, and F)
**Phylogenetic resolution between clades**	Poor	Poor	Poor	Average
**Phylogenetic resolution within clades**	Good (clade C)	Good	Good	Good
	Average (clades A, B)			
	Poor (clades D–H)			
**Potential for fine-scale analyses within clade C**	Yes	No (paralogous copies)	Yes	Yes

Cytochrome oxydase I (*coI*) is a key enzyme in aerobic metabolism in prokaryotes and eukaryotes [63] and is best known as the molecule used in barcoding a diversity of animals and other eukaryotes [64], including *Symbiodinium* [65]. This molecule has also been used in a previous phylogenetic reconstruction of *Symbiodinium* clades A–F [29]. This analysis revealed that the relationships among clades inferred using *coI* were mostly congruent with those obtained using nuclear and chloroplastic rDNA and that the gene yielded a relatively low level of resolution, a facet that likely explains why it has not been applied more broadly to the genus. Here, we obtained an unexpectedly high average genetic divergence in *coI* among clade C types (1.13%; Table 3), essentially driven by samples C90 and C91. These high levels of sequence divergence appear to be unique to clade C as distinct *ITS-2* types were indistinguishable in clades D, F, G, and H using this gene (Figure 3B, Figure S2). A more comprehensive analysis across a wider diversity of known clade C types will be needed to determine the usefulness of *coI* as a phylogenetic marker with utility for exploring patterns in diversity in clade C, however our initial results look very promising.

*Calmodulin* is a calcium-binding protein expressed in all eukaryotic cells. It can bind to and regulate a number of different protein targets, thereby affecting many different cellular functions [66], which is why it is common and highly expressed in dinoflagellate assemblages [53]. Despite appreciable levels of genetic divergences between clade C types using the exons regions of *calmodulin* (4.7% of averaged genetic divergence; Table 3), the presence of paralogous copies within clade C samples and/or additional samples deviating significantly from their expected phylogenetic position (clades A, B, C, F, G, and the outgroup *G. simplex*: see Figure S3) leads us to conclude that this gene has limited utility in *Symbiodinium* taxonomy.

The remaining two genes, *rad24* and *actin*, both contain introns and are both important components of cellular function and structure. *Rad24* is a DNA damage checkpoint protein that has been shown to promote the repair of double-stranded breaks during meiosis in yeast [67]. *Actin* proteins are major components of the cytoskeleton as well as mediators of internal cell motility [68]. Despite the presence of paralogous copies and/or intragenomic variants within clades A, B, and F, our results showed no evidence of paralogous copies within clade C samples (Figure 3D and 3E; Figures S4 and S5). In addition, the extremely low sequence variability between cloned sequences in the *Symbiodinium* types C1, C15, and/or C91 across both the exon and intron regions of these genes (data not shown) suggest that both *rad24* and *actin* warrant further investigation for *Symbiodinium* clade C.

### Conclusions

We employed a three-step procedure for identifying, characterizing and selecting novel *Symbiodinium* genes from cDNA libraries. Out of 84 candidate genes identified via multiple BLAST analyses of 6 dinoflagellate EST libraries, 7 *Symbiodinium* genes (*elf2*, *coI*, *coIII*, *cob*, *calmodulin*, *rad24*, and *actin*) were shortlisted based on our capacity to align DNA sequences of these genes and design genus-wide *Symbiodinium* PCR primers. These genes plus three benchmark genes (*ITS-2, nr28S and cp23S*) were amplified from 26 DNAs representing *22 Symbiodinium* types (clades A–H) and 3 dinoflagellates that served as outgroups. Four genes (*coI*, *calmodulin*, *rad24*, and *actin*) were selected for in-depth phylogenetic analyses based on their similar or higher rates of genetic divergences within *Symbiodinium* clade C, relative to the *ITS-2*. *Calmodulin*, *rad24*, and *actin* contained from three to twelve variable introns of potential interest for fine-scale analyses of *Symbiodinium*. Phylogenetic analyses revealed that *calmodulin* has limited taxonomic utility in *Symbiodinium* but that *coI*, *rad24*, and *actin* behave predictably with respect to *Symbiodinium* clade C specifically and are potentially excellent candidates as new markers for the field. These genes are currently the subjects of additional analysis exploring how intragenomically variable they are in single *Symbiodinium* cells. Our results clearly indicate that no single gene fits all of the taxonomic questions that we have for the genus *Symbiodinium*, and that extensive sequence analyses are required to validate new markers before they can be broadly applied. Additionally, the very limited number of *Symbiodinium* ESTs identified here as belonging to a ‘low expression category’, might have precluded us from selecting highly variable genes with low copy number [see 14]. Our approach, however, was effective in identifying new candidate genes for *Symbiodinium* and can serve as a model for future studies aimed at identifying novel genes from the massive transcriptomic datasets being generated from a wide range of taxa using Next Generation Sequencing technologies.

## Materials and Methods

### Identification of Candidate Genes

Eight expressed sequence tag (EST) libraries were obtained from the National Center for Biotechnology Information (NCBI) database (GenBank) in May 2007. The libraries represented *Symbiodinium* clade A (2,163 sequences); *Symbiodinium* clade C (5,156 sequences); *Amphidinium carterae* (3,383 sequences); *Alexandrium tamarense* (10,885 sequences); *Heterocapsa triquetra* (6,807 sequences); *Karenia brevis* (6,986 sequences); *Karlodinium micrum* (16,532 sequences) and *Lingulodinium polyedrum* (3,639 sequences). Each of these libraries differed widely. For example, the *Symbiodinium* clade A library was generated from cells that have been in cultures for over 25 years [37], whereas the clade C library encompasses *Symbiodinium* cDNAs isolated from the staghorn coral *Acropora aspera* exposed to a variety of stresses, including elevated temperature, ammonium supplementation, and seawater with different inorganic carbon concentrations [38]. The other dinoflagellate EST libraries were obtained from cultures grown and harvested under a variety of conditions, including isolation during different phases of growth or time points in the daily cycle [39]–[43].

Using the two *Symbiodinium* datasets as queries, a Perl script [44] linking the BLASTn output files from the BLAST v2.2.15 package (http://www.ncbi.nlm.nih.gov/) was used to retrieve homologous sequences from the six non-*Symbiodinium* dinoflagellate target libraries with an e-value threshold of 10^−25^. This relatively stringent cutoff was defined to restrict the integration of paralogous genes and limit the inclusion of short sequence fragments (<200 bp). Sequence identity of each homologous group of sequences was assessed at the protein-level using BLASTx. Eighty-four sequence alignments containing all homologous sequences retrieved in the BLAST analyses were created in the BioEdit v5.0.9 sequence alignment software [69] using ClustalW [70], then checked and manually edited. Because individual EST alignments contain sequences from either a single *Symbiodinium* clade (A or C) or both clades plus other dinoflagellates (see Table S1), candidate genes suitable for downstream characterization were selected using the following criterion: genes were shortlisted for gene characterization based on the presence of conserved regions that would allow for forward and reverse primer design. To facilitate work on all clades of *Symbiodinium*, alignments containing contigs from both *Symbiodinium* libraries (A and C) were prioritized. *Symbiodinium* clades A and C represent the most ancestral and derived *Symbiodinium* lineages, respectively, so primers targeting these very divergent clades would most likely also allow *Symbiodinium* from all other clades (B, D, E, F, G, H and I) to be recovered. Non-*Symbiodinium* sequences were also included in these alignments, because they provided information on how variable a given candidate gene was between dinoflagellate groups, while also allowing for the design of ‘*Symbiodinium*-specific’ primers in variable regions or ‘dinoflagellate-specific’ primers in conserved regions. In a single case where no *Symbiodinium* clade A contig was available for comparison with clade C (e.g. *calmodulin* gene; Table 1, Table S1), the non-*Symbiodinium* dinoflagellate contigs were used in the primer design. Finally, gene alignments were sorted again to identify those that allowed for design of primers yielding amplicons of between 150 bp and 1000 bp in length. Forward and reverse *Symbiodinium*-specific primers were designed across the conserved regions of the candidate genes using MacVector v11.0.2 (MacVector Inc., NC, USA), minimizing self/duplex hybridization and internal secondary structure problems.

### Characterization of candidate genes

Twenty-six DNA samples were used to generate sequences for phylogenetic analyses of the seven candidate genes (*elf2*, *coI*, *coIII*, *cob*, *calmodulin*, *rad24*, and *actin*) and three benchmark genes (*ITS-2*, *nr28S*, *cp23S*). The DNAs were extracted from fifteen *Symbiodinium* cultures belonging to five clades (A, B, D, E, and F); eight DNA samples belonging to four clades (C, F, G, and H) were isolated from symbiotic soritid foraminiferans harboring a single *ITS-2* symbiont type each [71]; and, the DNA samples representing the three dinoflagellate outgroups *Gymnodinium simplex* [CCMP 419], *Pelagodinium beii* [72], and *Polarella glacialis* [CCMP 1383]) were extracted from cultures (Table 2) according to Pochon et al. [73]. The recently described *Symbiodinium* clade I [17] was not analyzed here, but is currently being investigated in another multi-gene study that will be published elsewhere [Pochon et al. unpublished].

Gene fragments were PCR-amplified using the primer sets and annealing temperatures shown in Table S3 and 0.5 U Hotstart Immolase™ Taq polymerase (Bioline) in 50 µL reactions using the following thermocycling conditions: 95°C for 7 min followed by 38 cycles of 40 s at 94°C, 40 s at 52–58°C (see Table S3), 90 s at 72°C and a final extension at 72°C for 10 min. PCR products were purified using the QIAquick™ PCR Purification Kit (Qiagen). Two sequencing strategies were employed. First, purified products from all candidate genes were sequenced directly in both directions using the ABI Prism Big Dye™ Terminator Cycle Sequencing Ready Reaction Kit on an ABI 3100 Genetic Analyzer (Applied Biosystems) at the University of Hawai'i. Second, candidate genes that failed to provide high-quality direct sequences were cloned prior to sequencing. The gene products were ligated into the pGEM-T Easy vector™ (Promega), transformed into α-Select Gold Efficiency™ competent cells (Bioline) and grown overnight in Circlegrow® (MP Biomedicals). A minimum of five colonies were screened for inserts using plasmid-specific primers, and the positive screens were treated with exonuclease I and shrimp alkaline phosphatase and sequenced in both directions, as described above.

DNA sequences were inspected and assembled using Sequencher v4.7 (Gene Codes Corporation, Ann Arbor, MI, USA) and manually aligned with BioEdit v5.0.9. Cloned sequences were screened for potential PCR chimeras with the software package Bellerophon [45] and the chimeras removed from downstream analyses. The subclade identity of each *Symbiodinium* DNA sample was verified using local BLASTn against the *ITS-2* sequences in GeoSymbio_ITS2_LocalDatabase file, which is publicly available in GeoSymbio version 1.0.1 at https://sites.google.com/site/geosymbio/ [46].

Three sequence alignments were generated for each candidate gene. The first contained the genomic sequences amplified from DNA samples, and was used for downstream phylogenetic reconstructions (see below). The second contained both the genomic sequences amplified from DNA samples and the corresponding EST sequences. This alignment was used to identify the number and position of genomic introns, where applicable. The third alignment contained only clade C *Symbiodinium* sequences, and was used for calculating the genetic divergences among markers. For the latter, average uncorrected genetic distances between clade C sequence types (i.e., C1, C15, C90, and C91) for each candidate gene were calculated using the program Mega v4.0 [74]. Genetic distances for the intron-containing markers were calculated with and without introns. Whenever possible, the minimum, maximum and total variance values were indicated. A single sequence per clade C type was selected in the analysis, resulting in the incorporation of between 2 to 4 sequences per marker. For the markers that were cloned, the sequence showing the shortest branch length in each clade C type was selected (data not shown). In cases where several sequences showed the same short branch length, one sequence was randomly chosen among them and included in the analysis. Candidate genes displaying equal or higher than *ITS-2* genetic divergences within *Symbiodinium* clade C were selected for full phylogenetic reconstruction as detailed below.

### Gene Selection

Phylogenetic analyses were run on six genes; these included two *Symbiodinium* benchmark genes (*nr28S* and *cp23S*) and four candidate genes (*coI*, *calmodulin*, *rad24*, and *actin*). The goal of these analyses was to infer phylogeny for each gene, and compare the phylogenetic positions of *Symbiodinium* clades. Phylogenetic analyses for *calmodulin*, *rad24*, and *actin* were only performed on the exon regions of these genes (Figure 3; Figures S3, S4, S5) and each gene alignment was analyzed independently using Maximum-likelihood (ML) and Bayesian environments. Best-fit models of evolution and ML inferences with global tree searching procedure (10 starting trees) were estimated using TREEFINDER v12.2.0 [75]. Robustness of phylogenetic inferences was estimated using the bootstrap method [76] with 100 pseudoreplicates in all analyses. Bayesian analyses were performed using the parallel version of MrBayes v3.1.2 [77], starting from a random tree of 4 Metropolis-coupled Markov Chain Monte Carlo (MCMCMC), and including 1,000,000 generations with sampling every 10 generations. The average standard deviation of split frequencies was used to assess the convergence of the two runs. In all cases, the chains converged within 0.35×10^6^ generations. Therefore, the first 35,000 trees were discarded as burn-in and a 50% majority-rule consensus tree was calculated from the remaining 65,000 trees. Nodal support was reported as bayesian posterior probabilities.

## Supporting Information

Figure S1 **Phylogenies of** ***Symbiodinium*** **benchmark markers** ***nr28S*** **and** ***cp23S*** **.** Maximum likelihood (ML) phylograms of the genus *Symbiodinium* based on (**A**) 71 nuclear large subunit (*nr28S*) sequences, and (**B**) 71 chloroplastic large subunit (*cp23S*) sequences. Numbers at nodes represent the ML bootstrap support values (underlined numbers; 100 bootstrap pseudoreplicates performed) and Bayesian posterior probabilities. Black dots represent nodes with 100% bootstrap support and Bayesian posterior probabilities of 1.0. Nodes without numbers correspond to bootstrap supports and Bayesian posterior probabilities lower than 70% and 0.8, respectively. *Symbiodinium* clades are indicated with letters A to H, and each sequence is described by its *ITS-2* subclade name. GenBank accession numbers are given in brackets. Phylograms were rooted using either the dinoflagellates *Gymnodinium simplex*, *Pelagodinium beii*, and/or *Polarella glacialis*.
(TIF)

Figure S2 **Phylogeny of the** ***Symbiodinium*** **gene** ***coI*** **.** Best Maximum likelihood (ML) topology for *Symbiodinium* clades A to H based on 26 cytochrome oxidase subunit 1 (*coI*) sequences (alignment size: 1057 bp). Numbers at nodes represent the ML bootstrap support values (underlined numbers; 100 bootstrap pseudoreplicates performed) and Bayesian posterior probabilities. Black dots represent nodes with 100% bootstrap support and Bayesian posterior probabilities of 1.0. Nodes without numbers correspond to bootstrap supports and Bayesian posterior probabilities lower than 70% and 0.8, respectively. Nodes displaying bootstrap support values lower than 50% were manually collapsed. The phylogram was rooted using the dinoflagellates *Gymnodinium simplex*, *Pelagodinium beii*, and *Polarella glacialis*. GenBank accession numbers are given in brackets.
(TIF)

Figure S3 **Phylogeny of the** ***Symbiodinium*** **gene** ***calmodulin*** **.** Best Maximum likelihood (ML) topology for *Symbiodinium* clades A to H based on the exon regions of 92 *calmodulin* sequences (alignment size: 154 bp). Numbers at nodes represent the ML bootstrap support values (underlined numbers; 100 bootstrap pseudoreplicates performed) and Bayesian posterior probabilities. Black dots represent nodes with 100% bootstrap support and Bayesian posterior probabilities of 1.0. Nodes without numbers correspond to bootstrap supports and Bayesian posterior probabilities lower than 70% and 0.8, respectively. Nodes displaying bootstrap support values lower than 50% were manually collapsed. The phylogram was rooted using either the dinoflagellates *Gymnodinium simplex*, *Pelagodinium beii*, and *Polarella glacialis*. Paralogous copies shown in red and samples that deviate significantly from the expected phylogenetic position shown in blue. GenBank accession numbers are given in brackets.
(TIF)

Figure S4 **Phylogeny of the** ***Symbiodinium*** **gene** ***rad24*** **.** Best Maximum likelihood (ML) topology for *Symbiodinium* clades A to H based on the exon regions of 73 *rad24* sequences (alignment size: 580 bp). Numbers at nodes represent the ML bootstrap support values (underlined numbers; 100 bootstrap pseudoreplicates performed) and Bayesian posterior probabilities. Black dots represent nodes with 100% bootstrap support and Bayesian posterior probabilities of 1.0. Nodes without numbers correspond to bootstrap supports and Bayesian posterior probabilities lower than 70% and 0.8, respectively. Nodes displaying bootstrap support values lower than 50% were manually collapsed. The phylogram was rooted using the dinoflagellates *Gymnodinium simplex*, *Pelagodinium beii*, and *Polarella glacialis*. Paralogous copies shown in red; samples deviating significantly from the expected phylogenetic position shown in blue; and samples displaying both paralogous copies and significant deviation from expected phylogenetic position shown in green. GenBank accession numbers are given in brackets.
(TIF)

Figure S5 **Phylogeny of the** ***Symbiodinium*** **gene** ***actin*** **.** Best Maximum likelihood (ML) topology for *Symbiodinium* clades A to H based on the exon regions of 71 *actin* sequences (alignment size: 925 bp). Numbers at nodes represent the ML bootstrap support values (underlined numbers; 100 bootstrap pseudoreplicates performed) and Bayesian posterior probabilities. Black dots represent nodes with 100% bootstrap support and Bayesian posterior probabilities of 1.0. Nodes without numbers correspond to bootstrap supports and Bayesian posterior probabilities lower than 70% and 0.8, respectively. Nodes displaying bootstrap support values lower than 50% were manually collapsed. The phylogram is rooted using the dinoflagellates *Gymnodinium simplex*, *Pelagodinium beii*, and *Polarella glacialis*. Paralogous copies shown in red and samples that deviate significantly from the expected phylogenetic position shown in blue. GenBank accession numbers are given in brackets.
(TIF)

Table S1 **Complete list of (n = 84) candidate genes identified after BLASTn comparisons of eight dinoflagellate EST libraries.**
(PDF)

Table S2 **Models of evolution for phylogenetic inferences.** Best-fit models of evolution obtained in Treefinder, with corresponding gamma distribution (G) and proportion of invariant sites (I). Best-fit models were applied to each DNA alignments used in this study for phylogenetic reconstruction. The references indicate the original description of each model.
(PDF)

Table S3 **PCR primers.** *Symbiodinium* gene names, primer pairs, approximate base-pair distances between primers (based on the EST sequence alignments), and annealing temperatures (TM) used to amplify gene sequences for phylogenetic analysis.
(PDF)
