# Supplementary figures and images for: Identifying and Characterizing Alternative Molecular Markers for the Symbiotic and Free-Living Dinoflagellate Genus Symbiodinium
Source: PLoS One. 2012 Jan 4;7(1):e29816. doi: 10.1371/journal.pone.0029816 (PMC3251599; doi:10.1371/journal.pone.0029816)

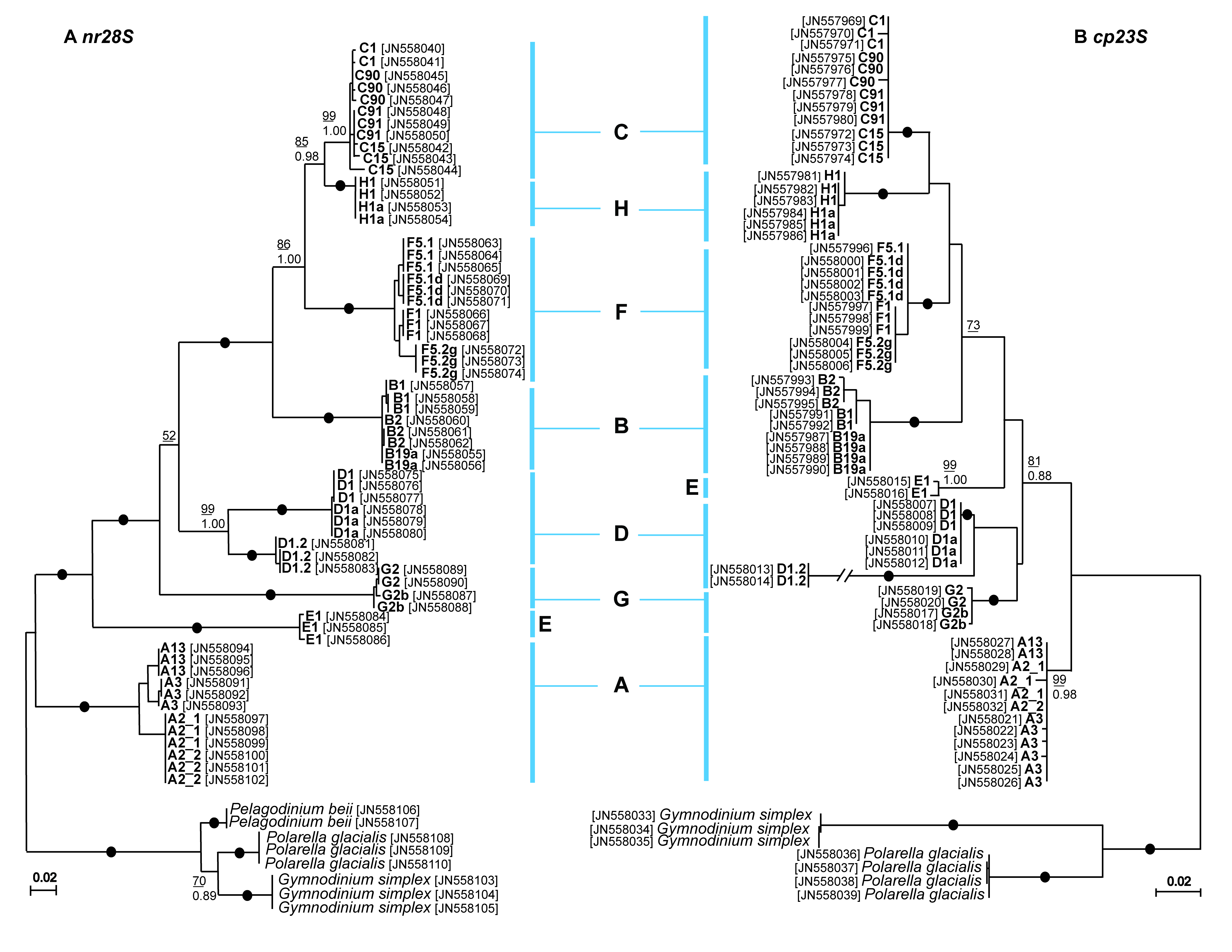

Supplement: Figure S1 — Phylogenies of Symbiodinium benchmark markers nr28S and cp23S . Maximum likelihood (ML) phylograms of the genus Symbiodinium based on (A) 71 nuclear large subunit (nr28S) sequences, and (B) 71 chloroplastic large subunit (cp23S) sequences. Numbers at nodes represent the ML bootstrap support values (underlined numbers; 100 bootstrap pseudoreplicates performed) and Bayesian posterior probabilities. Black dots represent nodes with 100% bootstrap support and Bayesian posterior probabilities of 1.0. Nodes without numbers correspond to bootstrap supports and Bayesian posterior probabilities lower than 70% and 0.8, respectively. Symbiodinium clades are indicated with letters A to H, and each sequence is described by its ITS-2 subclade name. GenBank accession numbers are given in brackets. Phylograms were rooted using either the dinoflagellates Gymnodinium simplex, Pelagodinium beii, and/or Polarella glacialis. (TIF) [file pone.0029816.s001.tif]

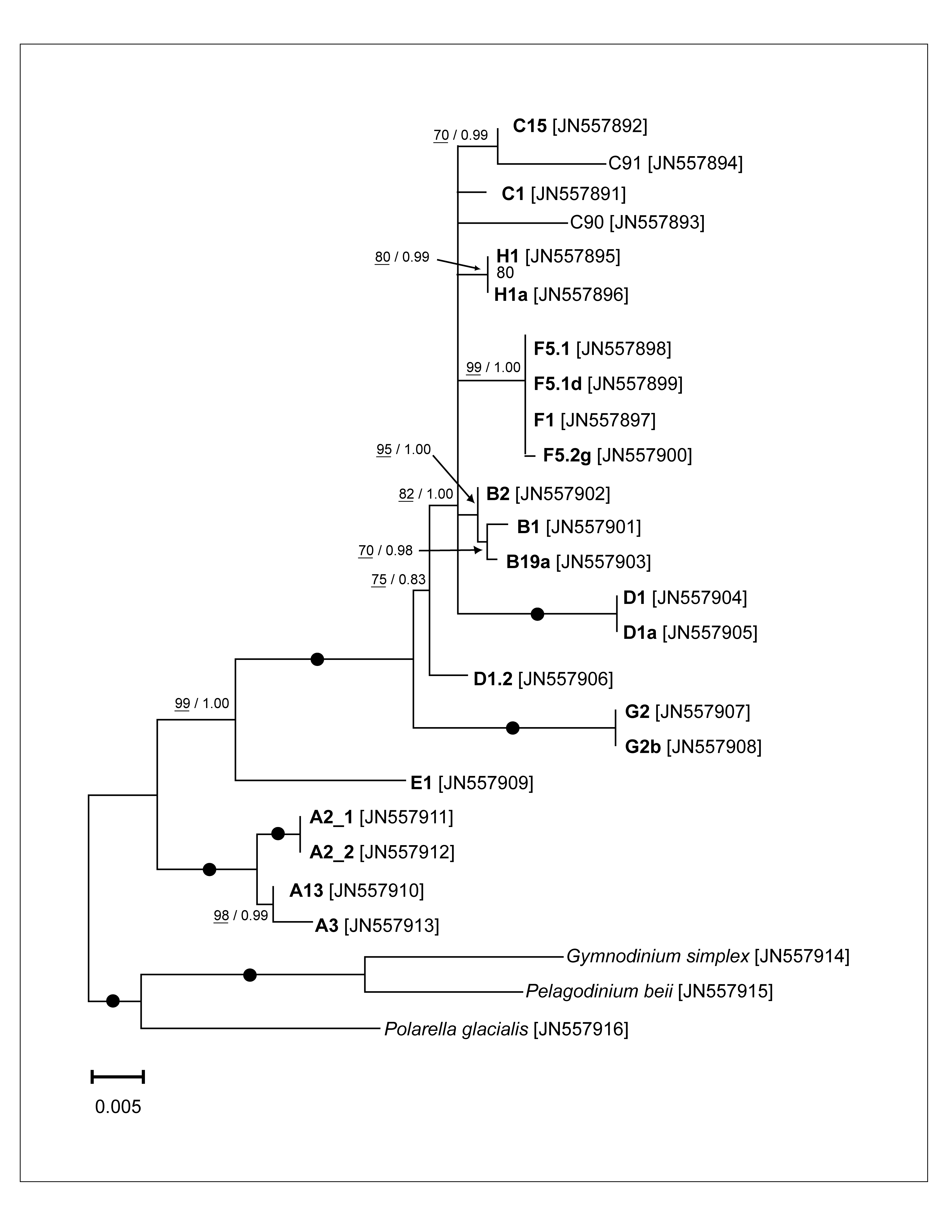

Supplement: Figure S2 — Phylogeny of the Symbiodinium gene coI . Best Maximum likelihood (ML) topology for Symbiodinium clades A to H based on 26 cytochrome oxidase subunit 1 (coI) sequences (alignment size: 1057 bp). Numbers at nodes represent the ML bootstrap support values (underlined numbers; 100 bootstrap pseudoreplicates performed) and Bayesian posterior probabilities. Black dots represent nodes with 100% bootstrap support and Bayesian posterior probabilities of 1.0. Nodes without numbers correspond to bootstrap supports and Bayesian posterior probabilities lower than 70% and 0.8, respectively. Nodes displaying bootstrap support values lower than 50% were manually collapsed. The phylogram was rooted using the dinoflagellates Gymnodinium simplex, Pelagodinium beii, and Polarella glacialis. GenBank accession numbers are given in brackets. (TIF) [file pone.0029816.s002.tif]

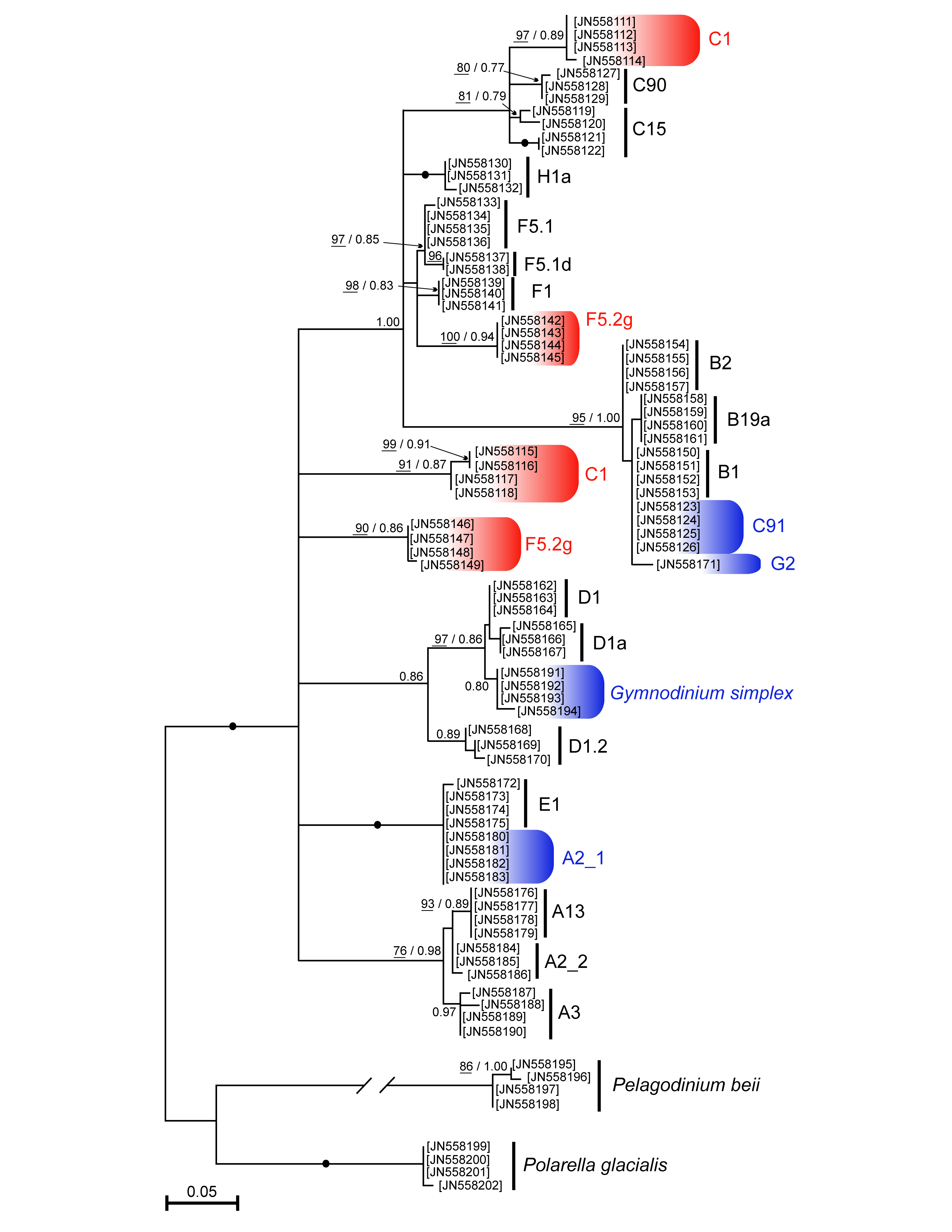

Supplement: Figure S3 — Phylogeny of the Symbiodinium gene calmodulin . Best Maximum likelihood (ML) topology for Symbiodinium clades A to H based on the exon regions of 92 calmodulin sequences (alignment size: 154 bp). Numbers at nodes represent the ML bootstrap support values (underlined numbers; 100 bootstrap pseudoreplicates performed) and Bayesian posterior probabilities. Black dots represent nodes with 100% bootstrap support and Bayesian posterior probabilities of 1.0. Nodes without numbers correspond to bootstrap supports and Bayesian posterior probabilities lower than 70% and 0.8, respectively. Nodes displaying bootstrap support values lower than 50% were manually collapsed. The phylogram was rooted using either the dinoflagellates Gymnodinium simplex, Pelagodinium beii, and Polarella glacialis. Paralogous copies shown in red and samples that deviate significantly from the expected phylogenetic position shown in blue. GenBank accession numbers are given in brackets. (TIF) [file pone.0029816.s003.tif]

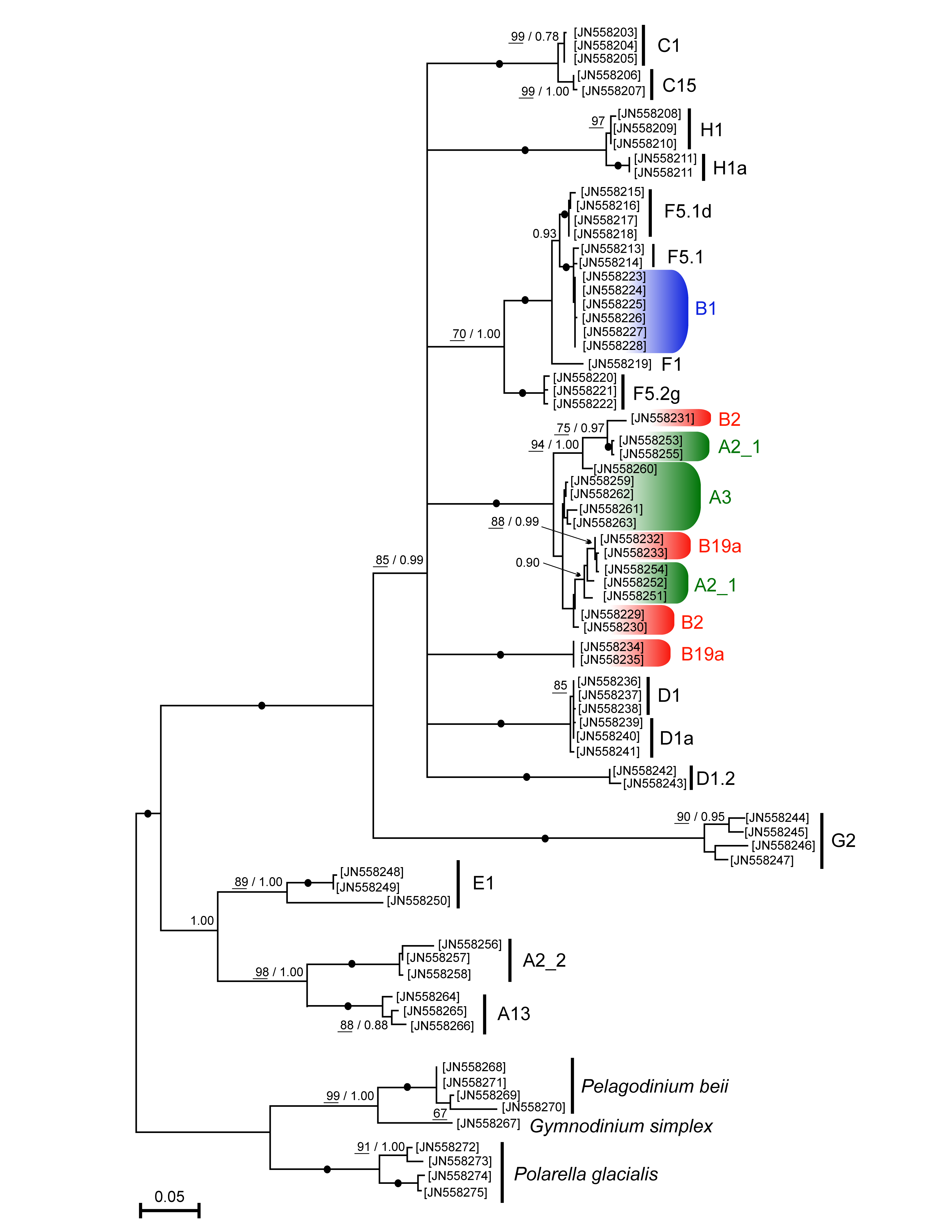

Supplement: Figure S4 — Phylogeny of the Symbiodinium gene rad24 . Best Maximum likelihood (ML) topology for Symbiodinium clades A to H based on the exon regions of 73 rad24 sequences (alignment size: 580 bp). Numbers at nodes represent the ML bootstrap support values (underlined numbers; 100 bootstrap pseudoreplicates performed) and Bayesian posterior probabilities. Black dots represent nodes with 100% bootstrap support and Bayesian posterior probabilities of 1.0. Nodes without numbers correspond to bootstrap supports and Bayesian posterior probabilities lower than 70% and 0.8, respectively. Nodes displaying bootstrap support values lower than 50% were manually collapsed. The phylogram was rooted using the dinoflagellates Gymnodinium simplex, Pelagodinium beii, and Polarella glacialis. Paralogous copies shown in red; samples deviating significantly from the expected phylogenetic position shown in blue; and samples displaying both paralogous copies and significant deviation from expected phylogenetic position shown in green. GenBank accession numbers are given in brackets. (TIF) [file pone.0029816.s004.tif]

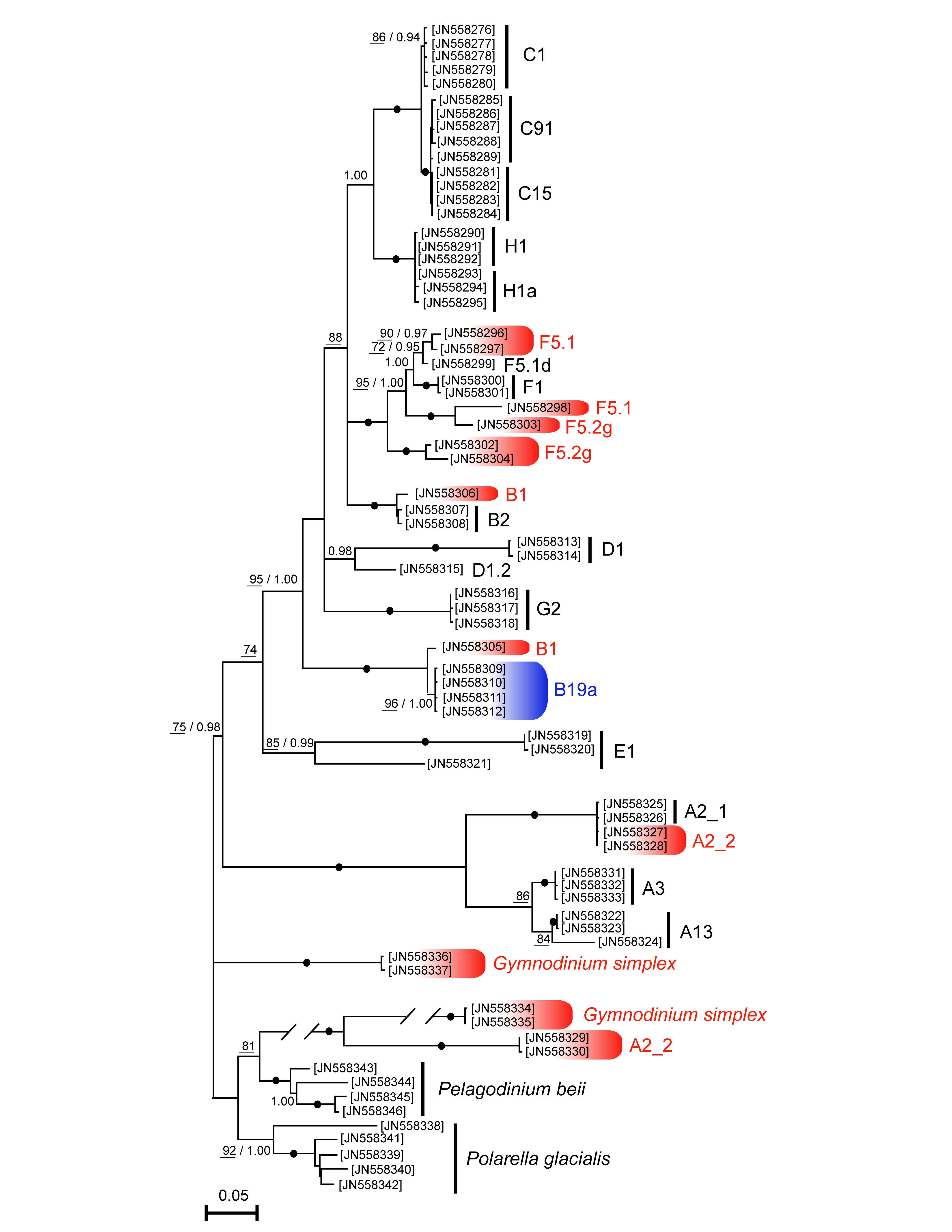

Supplement: Figure S5 — Phylogeny of the Symbiodinium gene actin . Best Maximum likelihood (ML) topology for Symbiodinium clades A to H based on the exon regions of 71 actin sequences (alignment size: 925 bp). Numbers at nodes represent the ML bootstrap support values (underlined numbers; 100 bootstrap pseudoreplicates performed) and Bayesian posterior probabilities. Black dots represent nodes with 100% bootstrap support and Bayesian posterior probabilities of 1.0. Nodes without numbers correspond to bootstrap supports and Bayesian posterior probabilities lower than 70% and 0.8, respectively. Nodes displaying bootstrap support values lower than 50% were manually collapsed. The phylogram is rooted using the dinoflagellates Gymnodinium simplex, Pelagodinium beii, and Polarella glacialis. Paralogous copies shown in red and samples that deviate significantly from the expected phylogenetic position shown in blue. GenBank accession numbers are given in brackets. (TIF) [file pone.0029816.s005.tif]
