# Supplementary material for: Identifying and Characterizing Alternative Molecular Markers for the Symbiotic and Free-Living Dinoflagellate Genus Symbiodinium
Source: PLoS One. 2012 Jan 4;7(1):e29816. doi: 10.1371/journal.pone.0029816 (PMC3251599; doi:10.1371/journal.pone.0029816)
Supplement: Table S1 — Complete list of (n = 84) candidate genes identified after BLASTn comparisons of eight dinoflagellate EST libraries. (PDF) [file pone.0029816.s006.pdf]

Protein description	# EST	Libraries Hits [e-value ≤ 10^{-25}]
Hypothetical bacterial-like (bacl-1) protein[a]	136	C, Ac
Glyceraldehyde-3-phosphate dehydrogenase	59	A, C, Kb
Peridinin chlorophyll-a binding protein	54	A, C
Hypothetical bacterial-like (bacl-2) protein	38	C, Ht
Cytochrome oxidase subunit 3 (*coIII*) *	35	A, C, At, Kb, Km, Lp
Heat shock Protein 90	25	C, Ac, Ht, Kb, Km, Lp
Actin *	19	A, C, Ac, At, Ht, Kb, Km, Lp
Beta-tubulin	19	C, Ac, Ht, Km
Translation elongation factor GTPase	17	C, At
Heat shock protein 70	15	C, Ac, At, Ht, Kb. Km, Lp
Light-harvesting chlorophyll a-c binding protein	15	C, Lp
Plastid photosystem II 12kD extrinsic protein	11	A, C
phosphoenolpyruvate/phosphate translocator	10	A, C
DNA damage checkpoint protein (*rad24*) *	10	A, C, At, Km
S-Adenosyl homocysteine hydrolase	9	A, C, Ac, At, Ht, Kb, Lp
Alpha-tubulin	9	C, At, Kb, Km
Mitochondrial cytochrome B (*cob*)*	8	A, C, Kb, Km, Lp
Pyridoxine biosynthesis protein	8	C, Ac, Ht, Lp
Translation elongation Factor 1	8	A, C, Ac, At, Ht, Lp
Calmodulin *	8	C, Ac, At, Ht, Km, Kb, Lp
Plastid fructose bisphosphate aldolase	7	A, C, Ac, At, Ht, Kb, Km, Lp
Phosphoserine aminotransferase	7	C, Ac
Ammonia permease	7	A, C
Nitrate transporter	7	A, C
Chloroplast photosystem II cytochrome c550	7	C, At
Hypothetical cask-interacting protein	7	C, At, Ht, Kb, Lp
Chloroplast 23S ribosomal RNA	6	A, C
Conserved hypothetical protein	6	C, At
Chloroplast ferredoxin-NADP reductase	5	C, At, Lp
Carbamoyl-phosphate synthase	5	C, At
Chloroplast cytochrome c6	5	C, Ht
cyclophilin-like protein	5	C, At, Ht, Km, Lp
Cytochrome oxidase subunit 1 (*coI*) *	4	A, C, Ac, Kb, Km, Lp
Beta-galactosidase	4	C, Ac
Translation elongation Factor 2 (*elf2*)*	4	A, C, At, Km
ATPase replication factor C	4	C, Lp
Flagellar associated protein	4	C, Lp
ADP-ribosylation factor	4	C, At
Plastid protein NAP50	4	A, C
Unknown Function	4	A, C, Ht, Lp
Unknown Function	4	A, C, Ht, Kb, Km, Lp
ADP-ribosylation factor	4	A, C, Ac, At
Putative Symbiodinium-specific Orthologs[b]	3	A, C
Ribonucleotide-diphosphate reductase	3	A, C, Km, Lp
Ribulose bisphosphate carboxylase, form II	3	C, Ht, Lp
Nuclear 18S, ITS, 28S ribosomal RNA	3	A, C, Km

Polyubiquitin	3	C, Ac, Km
Ubiquitin-fold modifier-conjugating enzyme	3	C, At
GTP-binding nuclear protein ran/tc4	3	C, At, Km
Chloroplast photosystem I subunit XI	3	A, C
Probable family finger-like Protein	3	A, C
Plastid Oxygen evolving enhancer	3	C, At, Lp
Chloroplast ATP synthase	2	C, Ac, At, Ht
Chloroplast cytochrome b6	2	C, Ac
Succinate dehydrogenase	2	C, At, Km
Chloroplast phosphoglycerate kinase precursor	2	C, At
Plastid geranylgeranyl reductase/hydrogenase	2	C, At, Ht
Probable serine protease	2	C, Ac
Chloroplast photosystem I subunit VII	2	C, Ac, At, Kb, Lp
Chloroplast photosystem I, subunit II	2	A, C
Hypothetical transporter protein	2	A, C
Hypothetical membrane protein	2	A, C
Hypothetical fumarate reductase	2	A, C, Ht
Hypothetical binding protein	2	A, C
Unknown Function	2	A, C
Unknown Function	2	C, Ac
Chloroplast light harvesting complex protein	2	C, Ht
ATP citrate lyase	1	C, Ht
Probable fumarate reductase	1	C, Ht, Lp
Cobalamin-dependent methionine synthase	1	C, Ht
Phosphoglycerate mutase 1	1	C, Ht
16S ribosomal RNA	1	C, Km
Translation elongation Factor 3	1	C, Lp
Large subunit Ribosomal protein RPL-27a	1	C, Km, Lp
Putative luminal-binding chaperone	1	C, At
26S proteasome regulatory subunit	1	C, At, Km
Cullin 1-like protein C	1	C, Lp
Peptidyl-prolyl cis-trans isomerase	1	C, Ht
Peroxiredoxin V protein	1	C, At
Ubiquitin-conjugating enzyme	1	C, At, Ht, Km, Lp
Probable d-galactonate transporter	1	C, Lp
LSM4-like U6 small nuclear RNA	1	C, Km
Unknown Function	1	C, Ht
Clathrin assembly protein	1	C, At, Lp

Genes are sorted by decreasing level of transcript abundance in the two *Symbiodinium* EST libraries combined. Details of the EST libraries displaying hits in BLASTn are indicated for each gene candidate by letters A, C, Ac, At, Ht, Kb, Km, and Lp, which correspond to EST libraries *Symbiodinium* A, *Symbiodinium* C, *Amphidinium carterae*, *Alexandrium tamarense*, *Heterocapsa triquetra*, *Karenia brevis*, *Karlodinium micrum*, and *Lingulodinium polyedrum*, respectively. Protein descriptions were obtained using BLASTx. [a]The most abundant transcript corresponds to ortholog bacl-1 (See [38] for more information). [b]This putative *Symbiodinium*-specific ortholog corresponds to the 'SS_ortholog1' [37]. *Genes shortlisted for downstream phylogenetic analyses (characterization of candidate genes; Table 1).
