# Supplementary material for: Identifying and Characterizing Alternative Molecular Markers for the Symbiotic and Free-Living Dinoflagellate Genus Symbiodinium
Source: PLoS One. 2012 Jan 4;7(1):e29816. doi: 10.1371/journal.pone.0029816 (PMC3251599; doi:10.1371/journal.pone.0029816)
Supplement: Table S2 — Models of evolution for phylogenetic inferences. Best-fit models of evolution obtained in Treefinder, with corresponding gamma distribution (G) and proportion of invariant sites (I). Best-fit models were applied to each DNA alignments used in this study for phylogenetic reconstruction. The references indicate the original description of each model. (PDF) [file pone.0029816.s007.pdf]

Dataset	Figure	Model	G	I	Reference (see below)
nr28S rDNA	Figure 3A; Figure S1	GTR	0.449	0.119	[1]
cp23S rDNA	Figure S2	TN	0.3486		[2]
coI	Figure 3B; Figure S3	GTR	0.2749		[1]
calmodulin	Figure 3C; Figure S4	HKY	0.1701		[3]
rad24	Figure 3D; Figure S5	J2 (TIM)	0.7432	0.3227	[4]
actin	Figure 3E; Figure S6	GTR	0.3884		[1]

1. Lanave C, Preparata G, Saccone C, Serio G (1984) A new method for calculating evolutionary substitution rates. J Mol Evol 20: 86-93.
2. Tamura K, Nei M (1993) Estimation of the number of nucleotide substitutions in the control region of mitochondrial DNA in humans and chimpanzees. Mol Biol Evol 10: 512-526.
3. Hasegawa M, Kishino H, Yano K (1985) Dating of the human-ape splitting by a molecular clock of mitochondrial DNA. J Mol Evol 22: 160-174.
4. Rodriguez F, Oliver JL, Marin A, Medina JR (1990) The general stochastic model of nucleotide substitution. J Theor Biol 142: 485-501.
