# Supplementary material for: Identifying and Characterizing Alternative Molecular Markers for the Symbiotic and Free-Living Dinoflagellate Genus Symbiodinium
Source: PLoS One. 2012 Jan 4;7(1):e29816. doi: 10.1371/journal.pone.0029816 (PMC3251599; doi:10.1371/journal.pone.0029816)
Supplement: Table S3 — PCR primers. Symbiodinium gene names, primer pairs, approximate base-pair distances between primers (based on the EST sequence alignments), and annealing temperatures (TM) used to amplify gene sequences for phylogenetic analysis. (PDF) [file pone.0029816.s008.pdf]

Region	Primer	Primer sequence (5' to 3')	Size (bp)	TM
5.8S, *ITS-2*, *nr28S* rDNA	ITS-Dino (for)	GTGAATTGCAGAACTCCGTG	~1000	52
	LO (rev)	GCTATCCTGAGRGAAACTTCG		
cp23S rDNA	23S4F (for)	GACGGCTGTAACTATAACGG	~600	53
	23S7R (rev)	CCATCGTATTGAACCCAGC		
elf2	EF_f1 (for)	GTGTGCAGTACCTGATTGAGAT	~500	55
	EF_r1 (rev)	CTCCTCCATCTTGCTGCCC		
coI	CoxI_f1 (for)	AAATTGTAATCATAAACGCTTAGG	~1000	55
	CoxI_r1 (rev)	GGCATAACATTAAATCCTAAGAA		
coIII	CoxIII_f2 (for)	GCTCTATTATTTGTATCCTTCTTTTGG	~230	58
	CoxIII_r1 (rev)	CGAAATTCTTTAATCTGCAAACTAAT		
cob	Cob_f1 (for)	TGAAATCTCATTTACAATCATATCCTT	~900	58
	Cob_r1 (rev)	CTACAGGAAATTGACCACCTATCC		
calmodulin	Calmo_f3 (for)	TGGCAACGGCTTCATCA	~150	52
	Calmo_r2 (rev)	CATCATCATCTTCACGAACTC		
rad24	Rad24_f2 (for)	CAACTTGCTGTCTGTGGC	~600	55
	Rad24_r1 (rev)	CTGRTCVGAGGTCCASAGSGT		
actin	Actin_f1 (for)	GGMATCATGGTBGGCATG	~900	54
	Actin_r1 (rev)	GGAGATCCACATCTGCTGGAA		
